# Human autoantibodies against the 54 kDa protein of the signal recognition particle block function at multiple stages

**DOI:** 10.1186/ar1895

**Published:** 2006-01-26

**Authors:** Karin Römisch, Frederick W Miller, Bernhard Dobberstein, Stephen High

**Affiliations:** 1 University of Cambridge, Cambridge Institute for Medical Research and Department of Clinical Biochemistry, Cambridge, UK; 2 Environmental Autoimmunity Group, National Institute of Environmental Health Sciences, National Institutes of Health, HHS, Bethesda, Maryland, USA; 3 Zentrum für Molekulare Biologie der Universität Heidelberg (ZMBH), Heidelberg, Germany; 4 Faculty of Life Sciences, University of Manchester, UK

## Abstract

The 54 kDa subunit of the signal recognition particle (SRP54) binds to the signal sequences of nascent secretory and membrane proteins and it contributes to the targeting of these precursors to the membrane of the endoplasmic reticulum (ER). At the ER membrane, the binding of the signal recognition particle (SRP) to its receptor triggers the release of SRP54 from its bound signal sequence and the nascent polypeptide is transferred to the Sec61 translocon for insertion into, or translocation across, the ER membrane. In the current article, we have characterized the specificity of anti-SRP54 autoantibodies, which are highly characteristic of polymyositis patients, and investigated the effect of these autoantibodies on the SRP function *in vitro*. We found that the anti-SRP54 autoantibodies had a pronounced and specific inhibitory effect upon the translocation of the secretory protein preprolactin when analysed using a cell-free system. Our mapping studies showed that the anti-SRP54 autoantibodies bind to the amino-terminal SRP54 N-domain and to the central SRP54 G-domain, but do not bind to the carboxy-terminal M-domain that is known to bind ER signal sequences. Nevertheless, anti-SRP54 autoantibodies interfere with signal-sequence binding to SRP54, most probably by steric hindrance. When the effect of anti-SRP autoantibodies on protein targeting the ER membrane was further investigated, we found that the autoantibodies prevent the SRP receptor-mediated release of ER signal sequences from the SRP54 subunit. This observation supports a model where the binding of the homologous GTPase domains of SRP54 and the α-subunit of the SRP receptor to each other regulates the release of ER signal sequences from the SRP54 M-domain.

## Introduction

Both secretory and membrane proteins destined for entry into the eukaryotic secretory pathway carry hydrophobic signal sequences that direct them to the endoplasmic reticulum (ER). The signal sequence is often located towards the amino terminus of the protein, and in the case of presecretory proteins it is proteolytically removed after targeting is completed [1]. These ER targeting signals are recognized and bound by a small ribonucleoprotein complex, the signal recognition particle (SRP), as soon as they emerge from the ribosome during protein synthesis [2-4]. This co-translational binding of SRP causes an arrest or retardation of translation that is relieved upon the interaction of the nascent chain/ribosome/SRP complex with the SRP receptor complex located in the ER membrane [3]. The binding of SRP to the SRP receptor allows the release of the signal sequence from SRP in a process that is dependent upon GTP binding and hydrolysis [3] and that also requires the presence of the Sec61 translocon [5]. Translation of the targeted nascent chain resumes and the free SRP can enter a new cycle of targeting, while the signal sequence inserts into the ER translocon and is cleaved on the luminal side of the membrane by signal peptidase when a suitable site is available [6]

Mammalian SRP consists of a 7S RNA and six proteins of molecular weight 9 kDa, 14 kDa, 19 kDa, 54 kDa, 68 kDa and 72 kDa [3]. The SRP 9 kDa and 14 kDa proteins form a heterodimer that is involved in the SRP-mediated translation arrest or retardation [3,7], while the SRP 19 kDa protein facilitates the binding of the 54 kDa subunit of the signal recognition particle (SRP54) to the 7S RNA [3,4]. The SRP54 subunit binds to ER signal sequences via its methionine-rich carboxy-terminal region (M-domain) and interacts with the SRP receptor complex via its central GTP binding domain [3,4]. This latter interaction means that the targeting of nascent polypeptides to the ER membrane is regulated by three GTPases; that is, by SRP54 and both the α-subunit and β-subunit of the SRP receptor [8,9].

Human autoantibodies often recognize epitopes that are conserved during evolution and are essential for the function of the autoantigen [10,11]. Previous studies have shown that human autoantibodies against SRP immunoprecipitate the 7S RNA and all the SRP protein subunits from HeLa cell extracts, but recognize predominantly the SRP54 subunit on immunoblots [12-14]. Anti-SRP autoantibodies occur almost exclusively in patients with polymyositis, a syndrome characterized by chronic muscle inflammation of unknown cause [12-14], and they seem to define a distinct phenotypic, genetic and epidemiologic subgroup of myositis patients [15,16]. In an attempt to better characterize anti-SRP54 autoantibodies, we have identified SRP54 epitopes of the anti-SRP autoantibodies present in sera from polymyositis patients and investigated the effects of these autoantibodies on SRP functions *in vitro*.

## Materials and methods

### Materials

The T7 RNA polymerase and restriction enzymes were obtained from Roche Diagnostics GmbH (Mannheim, Germany). ^35^S-Methionine was obtained from Amersham Buchler GmbH (Braunschweig, Germany). Cycloheximide, 7-methyl-guanosine 5'-monophosphate and puromycin were supplied by Sigma Chemical Co. (St Louis, MO, USA). 4-(3-Trifluoromethyldiazarino)benzoic acid was a gift from Dr Josef Brunner of the Swiss Federal Institute of Technology (Zürich, Switzerland).

### Antibodies

Rabbit antibodies against peptides or protein fragments derived from SRP54 (831, 901, 903, 907, 908, 981, 982) have been described previously [17]. Sera containing anti-SRP autoantibodies used in this study were obtained from patients with polymyositis and are a subset of those previously described [15].

### Affinity purification of human anti-SRP autoantibodies

The overexpression and purification of a fragment corresponding to SRP54 amino acids 1–166 has been described previously [17]. A 1.8 mg sample of the purified fragment was coupled to Affigel-10 resin (BioRad, Munich, Germany) according to the instructions of the manufacturer. Briefly, 1 ml antiserum 19-1 or antiserum 25-1 diluted 1:3 in PBS was incubated overnight with the affinity matrix at 4°C. Unbound material was recovered, and the matrix was washed with 40 volumes of PBS and eluted with 100 mM glycine-HCl, pH 2.5, at 10 ml/hour. The peak fractions were pooled, dialysed against 20 mM Hepes-KOH, pH 7.9, 250 mM potassium acetate and were concentrated by centrifugation through Centricon-10 filter units (Amicon, Witten, Gemany) to 1 mg/ml final protein concentration. The yield was about 250 μg/ml serum. The unbound fraction was also dialysed and the volume was reduced to 1 ml.

### Preparation of Fab fragments

Fractions enriched for IgG were prepared from the human sera by batch adsorption to DE52-Cellulose (Whatman, Dassel, Germany). One millilitre of serum was incubated with 2.5 ml DE52 equilibrated in 10 mM potassium phosphate, pH 7.8, for 2 hours at 4°C. Under these conditions, serum proteins with the exception of IgG bound to DE52. The unbound fraction contained approximately 95% pure IgG with transferrin as the major contaminant. The yield was 4 mg IgG/ml serum. Fab fragments were prepared from the unbound fraction by standard methods [18]. IgG and Fab fractions were dialysed into 20 mM Hepes-KOH, pH 7.9, 250 mM potassium acetate.

### SRP54 constructs

The canine SRP54 and its truncated derivatives used to determine the anti-SRP autoantibody epitopes have been described previously [17]. Plasmids containing the coding regions for the SRP54 N-domain (SRP54N domain) and SRP54 G-domain (SRP54G domain) were constructed by PCR. The initiating ATG for SRP54N was changed to an *Nco*I site and a stop codon was introduced at amino acid position 97. For SRP54G, amino acid 99 was changed to an initiating ATG, its surroundings changed to an *Nco*I site and a stop codon introduced in place of the codon for residue 295. The PCR products were gel-purified and subcloned into pET-8c [19].

### *In vitro *transcription and translation of SRP54 and its derivatives

Linearized DNA was transcribed *in vitro *and translated in the wheatgerm cell-free system for 60 minutes at 25°C [17]. Digestion of *in vitro *translated SRP54 with V8 (endoproteinase Glu-C; Roche Diagnostics, Mannheim, Germany) was as previously described [17].

### Immunoprecipitations

For immunoprecipitations under 'native' conditions, 3–10 μl *in vitro *translated material was diluted into 100 μl of 50 mM Tris-HCl, pH 7.5, 1% Nonidet-P40, 150 mM NaCl, 2 mM ethylenediamine tetraacetic acid, 20 μg/ml phenylmethylsulphonylfluoride. For denaturing conditions, samples in the same buffer plus 0.5% SDS were incubated at 95°C for five minutes and were subsequently diluted 1:10 in buffer without SDS. Samples were precipitated with 1 μl serum or with 1 μg IgG or Fab.

### *In vitro *translation and translocation of preprolactin

pSPBP4 contains the coding region for preprolactin (PPL) [20] and was a gift from Peter Walter (University of California, San Francisco, CA, USA). For translocation assays, full-length PPL was synthesized as a ^35^S-methionine-labelled precursor using a wheatgerm cell-free translation system supplemented with 0.06 OD_280 _units of salt-washed dog pancreas rough microsomes [21] and 20 nM canine SRP [22] for 1 hour at 25°C. One aliquot of the translocation reaction was analysed directly by SDS-PAGE, a further aliquot was digested with 0.3 mg/ml proteinase K (Boehringer Mannheim, Mannheim, Germany) for 10 minutes at 25°C and a third aliquot was treated with proteinase K in the presence of 0.5% Nonidet-P40 (Sigma). The proteinase K was quenched by precipitation with 10% trichloroacetic acid (final concentration) at 4°C for 30 minutes. To assay the effect of autoantibodies on SRP function, 3 μl of 250 nM SRP were incubated with 1 μl patient serum or 1 μg IgG or Fab in 20 mM Hepes-KOH, pH 7.9, 250 mM potassium acetate at 25°C for 30 minutes. Then 2 μl of this mixture were used for the *in vitro *translation/translocation of PPL (25 μl final volume).

### Photo-crosslinking assays

Ribosome bound fragments of the secretory protein PPL, an artificial integral membrane protein derived from the N-terminus of the invariant chain of the MHC class II complex, and the transmembrane domain of multiple colony-stimulating factor and chloroamphenicol transferase (IMC-CAT) were used to assay the signal-sequence binding and ER targeting functions of SRP [23,24]. In this study, an 86-residue N-terminal region of PPL (PPL_86_) and a 103-residue N-terminal region of IMC-CAT (IMC-CAT_103_) were synthesized *in vitro *as ^35^S-methionine-labelled polypeptides as previously described [24]. Translation was carried out in a wheatgerm cell-free translation system supplemented with 3.75 pmol ε-4-(3-trifluoromethyldiazarino)benzoyl-lysine tRNA per 25 μl reaction to incorporate photo-activatable crosslinking probes into the nascent polypeptides [24].

In order to assay for the effect of the autoantibodies on the signal-sequence binding ability of SRP, ribosome-arrested PPL_86 _complexes were synthesized in the absence of exogenous SRP [25]. Canine SRP (0.5 pmol) was incubated with 2 μl serum, IgG, Fab fragments, affinity-purified antibodies or supernatant from affinity purification for 15 minutes at 25°C, and this mixture was added to 30 μl PPL_86 _translation mixture and incubated for a further five minutes at 25°C. The reaction mixture was cooled to 0°C and was subsequently UV irradiated as described elsewhere [25]. After irradiation the crosslinked products were precipitated with trichloroacetic acid and were analysed by SDS-PAGE.

To determine the effect of the autoantibodies on targeting to the ER membrane, the IMC-CAT_103 _polypeptide was synthesized in the presence of SRP to give a stable ribosome/nascent chain/SRP complex [24]. Two microlitres of serum, IgG, Fab fragments, affinity-purified antibodies or supernatant from affinity purification were added to 30 μl aliquots of the translation mixture. The samples were incubated for 15 minutes at 25°C and then 0.1 OD_280 _units of salt-washed rough microsomes were added to each. Samples were incubated for 5 minutes at 25°C, cooled to 0°C and UV irradiated as already described. After irradiation, the membranes were separated from the cytosolic fraction by centrifugation through a high-salt sucrose cushion. Thirty-microlitre samples were layered over a 150 μl cushion of 0.25 M sucrose, 50 mM Hepes-KOH, pH 7.5, 0.5 M KCl, 5 mM MgCl_2 _and the membranes were recovered by centrifugation in a Beckmann airfuge [24]. The supernatant was removed and precipitated with trichloroacetic acid prior to analysis by SDS-PAGE. The membrane pellet was solubilized in sample buffer and analysed by SDS-PAGE. All samples were resolved on 10–15% SDS polyacrylamide gradient gels and were subjected to fluorography prior to analysis.

## Results

### Human anti-SRP autoantibodies inhibit SRP-dependent protein translocation into the ER

SRP binds to ribosomes in such a way that SRP54 scans the emerging nascent chains for the presence of a signal sequence [2,26]. Once a signal sequence has been bound, further elongation is retarded until contact is made between SRP54 and the SRP receptor complex in the ER membrane. In order to better characterize functionally important regions of SRP54 *in situ*, we tested the effects of various sera containing antibodies specific for the SRP54 protein upon co-translational SRP-dependent targeting to the ER membrane.

**Figure 1:**
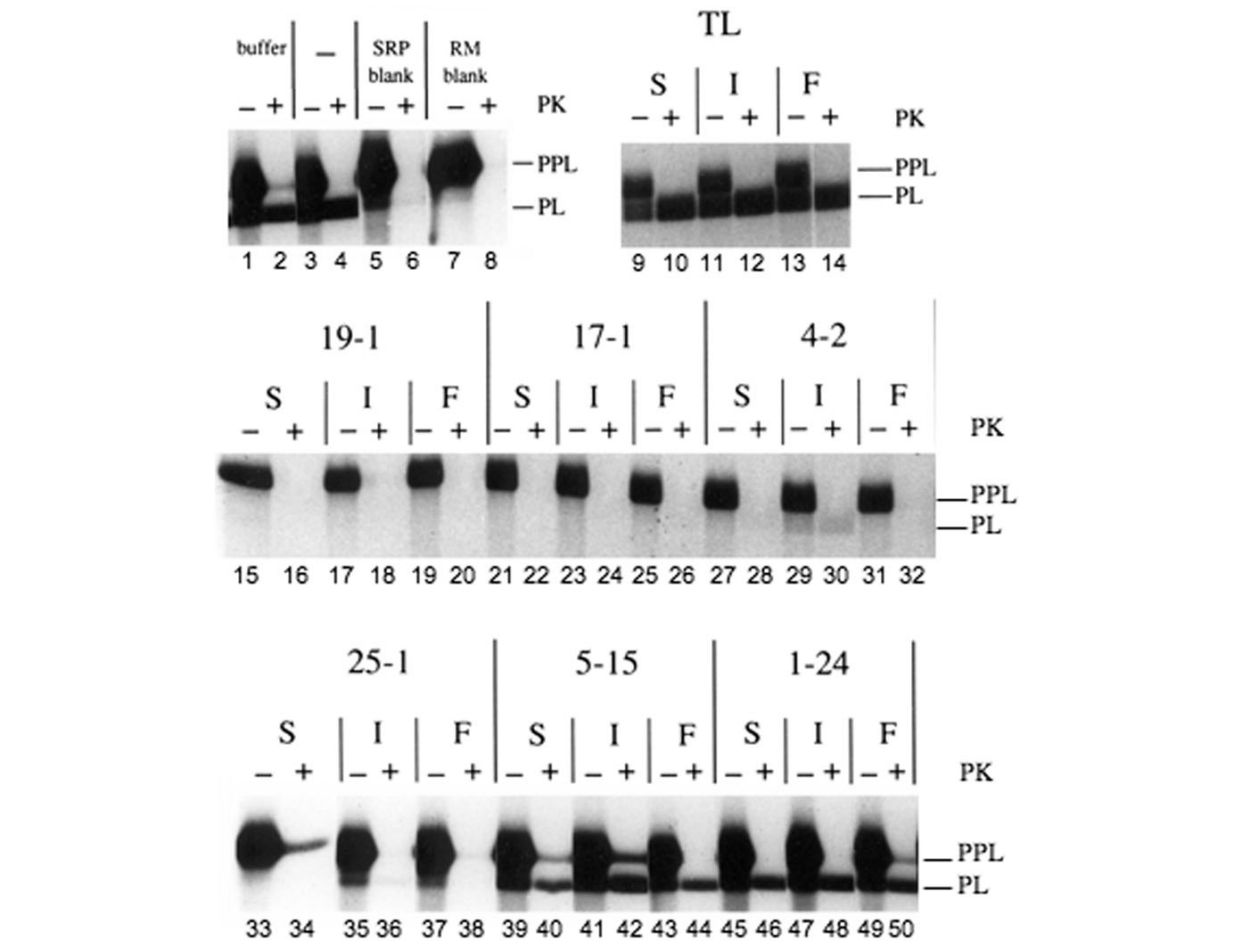
**Human sera containing autoantibodies directed against SRP inhibit protein translocation into the ER *in vitro***. The secretory precursor preprolactin (PPL) was synthesized as a ^35^S-radiolabelled protein using a cell-free system supplemented with signal recognition particle (SRP)-depleted endoplasmic reticulum (ER) membranes and purified SRP that had been preincubated with either buffer (lanes 1 and 2) or with no additions (lanes 3 and 4) to establish normal levels of prolactin (PL) translocation into ER-derived microsomes. The specificity of protein translocation was controlled for by performing experiments lacking exogenous SRP (lanes 5 and 6) or lacking salt-washed rough microsomal membranes (RM) (lanes 7 and 8). The complete translocation of signal-sequence-processed PL into the lumen of the ER microsomes was confirmed by showing resistance to digestion by proteinase K (cf. - and + PK). To investigate the ability of distinct autoantibodies to block function, SRP was preincubated with various anti-SRP-positive sera (S), IgG (I) or Fab (F) fractions prior to protein synthesis. TL, human control serum from a healthy individual, while 19-1, 17-1, 4-2 and 25-1 are human sera containing anti-SRP autoantibodies from polymyositis patients. Serum 5–15 is from a polymyositis patient without detectable myositis autoantibodies, and serum 1–24 is from a polymyositis patient with autoantibodies directed against histidyl-tRNA synthetase. PPL, unprocessed (signal-sequence containing) preprolactin that has not been translocated into the ER microsomes. PL, fully translocated, signal-cleaved prolactin located inside ER microsomes. Samples were analysed by SDS-PAGE on 10–15% gels and by fluorography.

In the first instance, we investigated the effect of these sera on the translocation of the secretory protein PPL into ER-derived microsomes using a cell-free translation system. PPL was synthesized in a wheatgerm system supplemented with salt-washed canine rough microsomes and SRP. Under standard conditions this leads to a significant level of protein translocation into the ER-derived microsomes, as evidenced by the appearance of prolactin (PL) from which the signal sequence had been cleaved (Figure 1, lanes 1 and 3). Signal-sequence cleavage occurs in the ER lumen, suggesting that the processed material had been translocated into the microsomal membranes. This was confirmed by establishing that the translocated PL chains were protected from digestion by exogenously added proteinase K, and hence were enclosed inside the microsomal membranes (Figure 1, lanes 2 and 4). In contrast, any remaining PPL from which the signal sequence had not been cleaved was digested by proteinase K treatment since it had not been translocated across the microsomal membranes (Figure 1, lanes 2 and 4).

The role of canine SRP in mediating PPL translocation in this cell-free system was confirmed by showing that no signal-sequence-processed or protease-protected PL chains were obtained when exogenous SRP was absent from the reaction mixture (Figure 1, lanes 5 and 6). Hence, the salt-washing had effectively removed all membrane-associated SRP from the microsomal membrane preparation [27]. Likewise, no PL chains were detected in the absence of added microsomal membranes (Figure 1, lanes 7 and 8).

In order to investigate the ability of anti-SRP54 antibodies to block SRP function, canine SRP was preincubated with various sera containing different anti-SRP autoantibodies prior to carrying out identical translocation assays using ER-derived microsomes. While the autoantisera clearly recognize human SRP54, it has been established that the amino acid sequences of the human and canine SRP54 subunits are identical [28]. Hence, the autoantisera should recognize the canine SRP54 subunit equally well. A number of antisera have been raised against synthetic peptides and recombinant fragments SRP54 in animals [17]; however, none of these had any effect upon PPL translocation in the assay outlined (data not shown). In contrast, the preincubation of SRP with human sera containing autoantibodies against SRP proteins from a number of polymyositis patients (19-1, 17-1, 4-2 and 25-1) completely abolished the translocation of the secretory protein precursor PPL into ER-derived microsomes (Figure 1, cf. lanes 15, 16, 21, 22, 27, 28, 33 and 34). No processing of PPL to PL by the luminal signal peptidase was therefore observed (Figure 1, lanes 15, 21, 27 and 33) and no protease-protected PL chains were seen after treatment with proteinase K (Figure 1, lanes 16, 22, 28 and 34).

That these effects were due to anti-SRP autoantibodies rather than other serum components was supported by the finding that preincubation with sera from a normal human control or from polymyositis patients containing either no detectable myositis autoantibodies (serum 5–15) or with anti-Jo-1 autoantibodies directed against histidyl-tRNA synthetase (serum 1–24) had little or no effect on PPL translocation (Figure 1, lanes 9, 39 and 45), and the finding that protease-protected PL was observed in all cases (Figure 1, lanes 10, 40 and 46). The specificity of these effects was underlined by the observation that both purified IgG fractions and monovalent Fab fragments gave almost identical results to those seen with the crude sera from which they were derived.

### Characterization and affinity purification of anti-SRP autoantibodies

**Figure 2:**
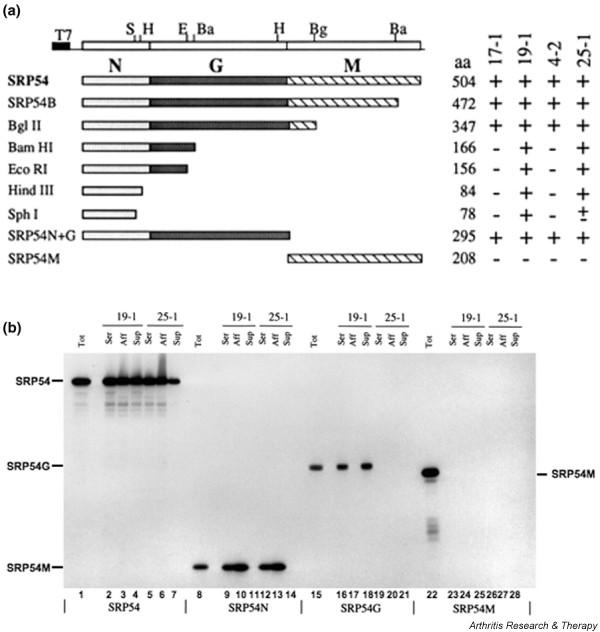
**Characterization of the regions of SRP54 recognized by the human autoantibodies. (a) **Summary of the immunoprecipitation of SRP54 and its fragments by the autoantibodies. A restriction map of the plasmid encoding SRP54 is shown at the top (cf [17]): S, *SphI*; H, *Hind*III; E, *Eco*RI; Ba, *Bam*HI; Bg, *Bgl*II. The complete SRP54 protein and truncated derivatives are outlined underneath. The names of the polypeptides are shown on the left, and their size in amino acids and the immunoreactivity with the patient autoantibodies is indicated on the right. **(b) **Immunoprecipitation of SRP54 and its separate domains by sera 19-1 and 25-1 autoantibodies affinity-purified on an amino-terminal SRP54 fragment (amino acids 1–166). SRP54, SRP54N, SRP54G and SRP54M domains were synthesized as ^35^S-radiolabelled polypeptides *in vitro *and an aliquot loaded directly onto the gel (Tot). Other aliquots were immunoprecipitated under native conditions with 1 μl serum (Ser), with 1 μl flow-through from the affinity column (Sup) or with 1 μg affinity-purified IgG (Aff). Samples were analysed by SDS-PAGE on 10–15% gels and by fluorography. The estimated molecular masses of the proteins were 54 kDa (SRP54), 23 kDa (SRP54G domain), 21 kDa (SRP54M domain) and 12 kDa (SRP54N domain).

As judged by immunoblotting, the anti-SRP autoantibodies from a number of patients react with SRP54 and the SRP 68 kDa and 72 kDa proteins – only serum 17-1 appeared to recognize solely SRP54 [14]. Immunoprecipitation from a mixture of *in vitro *translated SRP54 and the SRP 68 kDa and 72 kDa proteins suggested that the major fraction of each of the autoantibodies is directed against SRP54 (data not shown). In order to characterize the region of SRP54 recognized by the various autoantibodies, immunoprecipitations of a series of *in vitro *synthesized SRP54 fragments were performed. The results were identical with or without prior SDS denaturation, and are presented schematically in Figure 2a. On the basis of this analysis, we conclude that sera 4-2 and 17-1 recognize a region within the carboxy-terminal 130 amino acids of the SRP54G domain, while sera 19-1 and 25-1 recognize a region within the SRP54N domain. The epitope for 19-1 seems to be located more towards the N-terminus of this region than that of 25-1, since serum 19-1 precipitates the *Hind*III and *Sph*I derived fragments of SRP54 equally well whereas serum 25-1 reacts less well with the shorter *Sph*I fragment (Figure 2a). It is worth noting that none of the patient autoantibodies tested recognized the C-terminal SRP54 M-domain (SRP54M domain) (Figure 2a).

In order to establish whether the inhibitory effect of the human autoantibodies upon PPL translocation was a direct consequence of their anti-SRP54 activity, and to investigate whether antibodies recognizing different domains of SRP54 had different effects upon SRP function, specific antibodies were affinity-purified from sera 19-1 and 25-1. This was achieved using a 166-residue long N-terminal fragment of SRP54 that is equivalent to the N-domain plus a part of the G-domain (see [17]; Figure 2a, *Bam*HI derived fragment). The unbound fraction of the sera was also retained to establish whether it contained any distinct SRP54 reactive antibodies as a result of multiple activities being present in a single serum. Specificity was determined by immunoprecipitation of *in vitro *synthesized SRP54 fragments.

For both sera 19-1 and 25-1, the affinity-purified antibodies and the unbound fraction immunoprecipitated full-length SRP54 at a comparable level with the original sera (Figure 2b, lanes 2–7). When the SRP54N domain was examined, however, only the original sera and the affinity-purified antibodies reacted with this region (Figure 2b, lanes 9, 10, 12 and 13) while the unbound serum-derived fractions did not (Figure 2b, lanes 11 and 14). The affinity purification therefore specifically recovered autoantibodies recognizing the SRP54N domain while the unbound fraction was completely depleted of this activity. The specificity of the affinity-purified autoantibodies was further supported by the observation that neither preparation recognized the SRP54G domain (Figure 2b, lanes 17 and 20). The unbound material recovered from serum 19-1 did immunoprecipitate the SRP54G domain (Figure 2b, lane 18), while the equivalent fraction from serum 25-1 did not immunoprecipitate (Figure 2b, lane 21). We conclude that serum 19-1 contains at least two different antibody species, one recognizing the SRP54N domain within its N-terminal 78 residues (Figure 2a, *Sph*I fragment and Figure 2b, SRP54N reactivity of affinity-purified antibodies) and the second specific for a portion of the SRP54G domain between residues 166 and 295 (Figure 2a, SRP54N+G and Figure 2b, SRP54G reactivity of unbound fraction).

**Figure 3:**
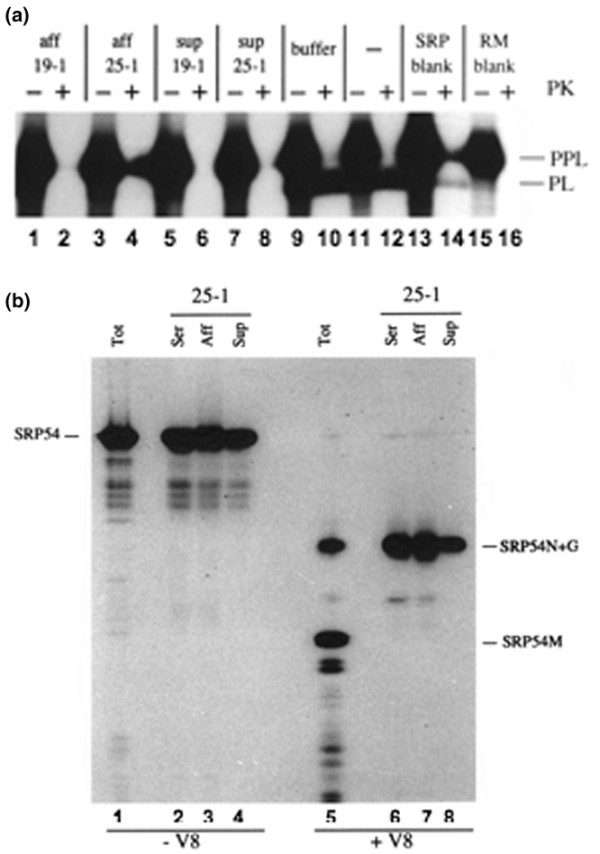
**Distinct autoantibodies against the SRP54N and SRP54G domains can both inhibit secretory protein translocation into the ER. (a) **The secretory protein preprolactin (PPL) was synthesized as a ^35^S-radiolabelled precursor *in vitro *in the presence of salt-washed rough microsomes (RM) and signal recognition particle(SRP) that was preincubated with affinity-purified autoantibodies from sera 19-1 or 25-1 on SRP54 amino acids 1–166 (Aff), with the flow-through fractions (Sup) or with buffer alone. SRP without preincubation (lanes 11 and 12) and translations without added SRP (lanes 13 and 14) or RM (lanes 15 and 16) were used as positive and negative controls, respectively. Samples were treated with proteinase K or not (+ or - PK) and were analysed by SDS-PAGE on 10–15% gels and by fluorography. **(b) **Serum 25-1 contains two distinct anti-SRP54 activities. SRP54 was synthesized as a ^35^S-radiolabelled protein *in vitro *and either digested with V8 protease (+ V8) or incubated in the absence of protease (- V8) as described previously [17]. An aliquot of both digested and undigested material was loaded onto the gel directly (Tot). Both digested and undigested material were immunoprecipitated using 1 μl serum 25-1 (Ser), using 1 μl affinity-column flow-through (Sup) or using 1 μg affinity-purified autoantibodies from serum 25-1 (Aff). Samples were analysed by SDS-PAGE on 10–15% gels and by fluorography.

We next established whether the effect of the autoantisera upon the SRP-dependent targeting reaction could be reproduced using purified autoantibodies. When the effect of the affinity-purified autoantibodies was examined, both the sera 19-1 and 25-1 derived samples were shown to block PPL translocation into rough microsomes as previously observed with the original sera (Figure 3, lanes 1–4). We therefore conclude that autoantibodies specific for the SRP54N domain can inhibit SRP function in an *in vitro *system. In both cases the unbound fraction also blocked SRP function (Figure 3, lanes 5–8), however, indicating that other activities may contribute to the effect of the original sera. In the case of 19-1, this may be attributed to the second SRP54G domain-reactive component of the serum we defined earlier. For serum 25-1, however, the unbound fraction was depleted of any detectable SRP54N reactive antibodies (see Figure 2b, lane 14) and showed no reaction with either SRP54G or SRP54M (Figure 2b, lanes 21 and 28).

One possibility was that serum 25-1 contained a second SRP54-reactive autoantibody population that recognized an epitope near the boundary of two of the SRP54 domains such that this was absent when they were analysed in isolation (cf. Figure 2b). To address this issue, SRP54 was treated with V8 protease to generate an intact SRP54N+G domain and a separate SRP54M domain [17]. The resulting products were then analysed by immunoprecipitation, and in this case we found that the SRP54N+G domain was efficiently recognized by the unbound serum 25-1 derived fraction following affinity purification (Figure 3b, lane 8). Thus, as is the case for serum 19-1, we conclude that 25-1 contains at least two distinct autoantibody populations that recognize distinct regions within the SRP54 protein.

### Anti-SRP autoantibodies interfere with signal-sequence binding of SRP54

**Figure 4:**
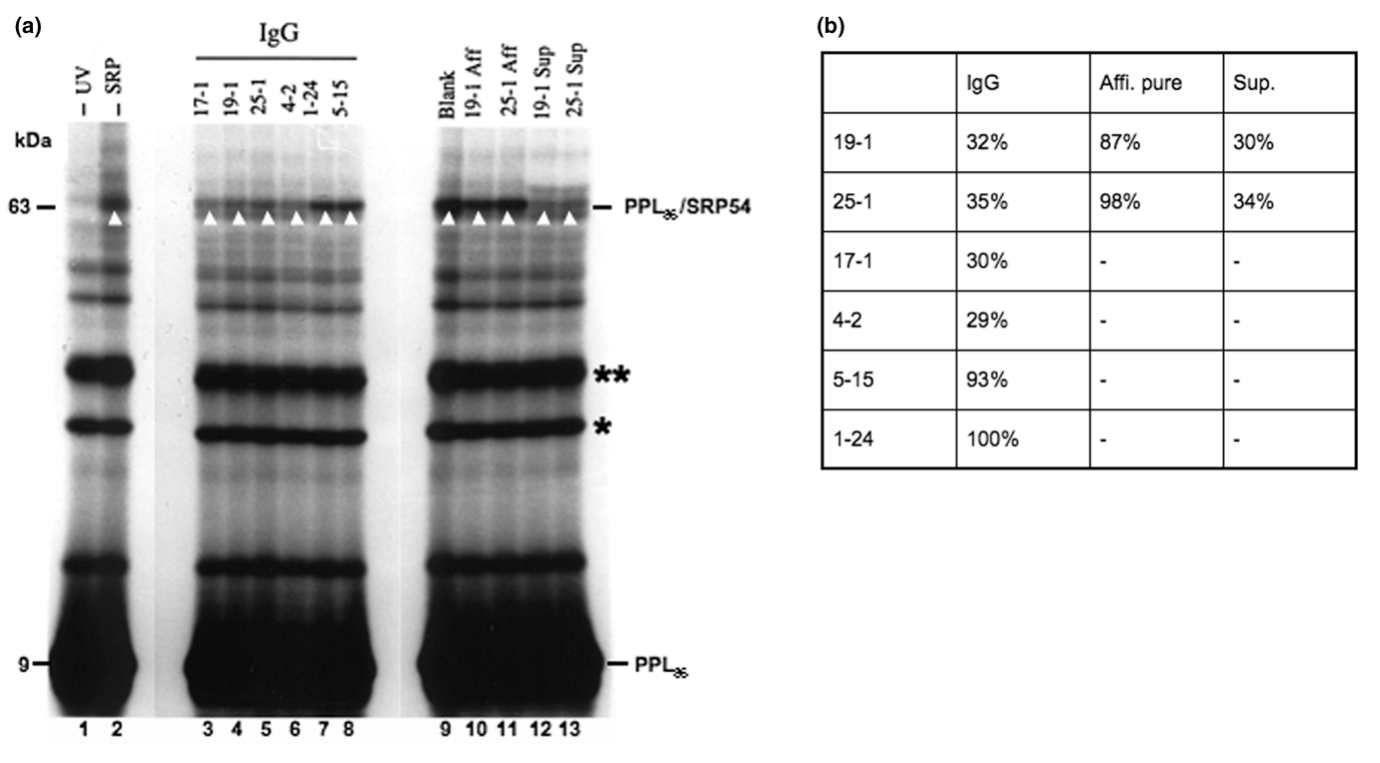
**Human autoantibodies against the SRP54G domain inhibit crosslinking of SRP54 to the PPL signal sequence. (a) **Canine SRP was preincubated with no additions (lane 9), with IgG fractions containing anti-SRP autoantibodies (lanes 3 to 8), with affinity-purified autoantibodies (Aff, lanes 10 and 11) or with their accompanying unbound fractions (Sup, lanes 12 and 13), and subsequently incubated with a ^35^S-radiolabelled fragment of preprolactin (PPL) present in the form of a ribosome nascent chain complex (PPL_86_). The interaction of the SRP54 subunit with the signal sequence of PPL_86 _was determined by UV-induced crosslinking. A non-irradiated sample (lane 1) and a sample lacking exogenously added SRP (lane 2) are shown as controls. The ~63 kDa crosslinking product (PPL_86_/SRP54) resulting from the crosslinking of the 86-residue PPL fragment to the 54 kDa subunit of canine SRP is indicated by a white arrowhead. A faint ~61 kDa product is visible in some lanes, especially where the binding of the canine SRP is strongly inhibited. This smaller species represents crosslinking of the nascent chain to the endogenous wheatgerm SRP54 homologue, and this product is normally only observed in the absence of canine SRP [34]. The ~9 kDa PPL_86 _fragment is indicated, as are the locations of full-length preprolactin (**) resulting from incomplete linearization of the DNA template for transcription of PPL_86_, and a peptidyl-tRNA species (*) resulting from the incomplete hydrolysis of the PPL_86_-tRNA bond during sample preparation. **(b) **The intensity of the 63 kDa band in (a) was quantified by scanning with an LKB Ultroscan XL enhanced laser densitometer. The most intense band achieved with a control serum or blank in each set (for instance, lanes 3–8 and lanes 9–13) was set to 100%.

Having obtained clear evidence that autoantibodies recognizing SRP54 could interfere with the SRP-dependent targeting process, we investigated the stage of the pathway at which targeting was inhibited. The first step required for SRP-dependent translocation of a nascent polypeptide chain across the ER membrane is the binding of its signal sequence to SRP54. The interaction between a signal sequence and SRP54 can be directly assessed by crosslinking [29]. We investigated the influence of preincubation of SRP with human anti-SRP autoantibodies upon its ability to bind to the PPL signal sequence using a well-established photo-crosslinking assay as a readout. When SRP54 is correctly bound to the PPL signal sequence, a radiolabelled, UV-dependent, photo-crosslinking product of 63 kDa is seen (Figure 4a, cf. lanes 1 and 2, PPL_86_/SRP54 indicated by white arrowhead; see also [25]). We found that the IgG fractions from polymyositis patients containing anti-SRP54 autoantibodies (sera 17-1, 19-1, 25-1, 4-2), but not from polymyositis patients without myositis autoantibodies or with other specificities (sera 1–24, 5–15), significantly reduced the crosslinking of SRP54 to the PPL signal sequence (Figure 4a, lanes 3–8, PPL_86_/SRP54, white arrowheads). When the levels of adduct formation were quantified, we discovered an approximately threefold reduction in crosslinking to SRP54 (Figure 4b). The crude sera and Fab preparations from each of these samples gave similar results (data not shown).

When the affinity-purified autoantibodies specific for the SRP54N domain were analysed, it was striking that they had no substantial effect upon the binding of SRP54 to the PPL signal sequence (Figure 4a, lanes 10 and 11, PPL_86_/SRP54, white arrowheads). In contrast, the unbound fractions derived from these preparations gave a level of inhibition comparable with the IgG fractions (Figure 4a, lanes 4, 5, 12 and 13, PPL_86_/SRP54, white arrowheads; see Figure 4b for quantification). Taken together, these data suggest that autoantibodies specific for the SRP54G domain (serum 19-1) and the SRP54N/G boundary (serum 25-1) block SRP binding to the PPL signal sequence *in vitro*. These data also suggest that any effect of the affinity-purified antibodies specific for the SRP54N domain upon SRP function (Figure 3a) must occur at a point after signal-sequence binding has occurred.

### Anti-SRP autoantibodies inhibit targeting to the ER membrane and the release of SRP54 from the signal sequence

Following the binding of SRP to a signal sequence present on a nascent polypeptide, the next step during ER targeting is the association of the ribosome/nascent chain/SRP complex with the SRP receptor complex [3]. This interaction leads to the release of the signal sequence from SRP54, allowing the polypeptide to interact with the ER translocation machinery [30]. In order to investigate the influence of the autoantibodies on this targeting step, we used a well-characterized, ribosome-associated, membrane protein fragment (IMC-CAT_103_) to generate stable ribosome/nascent chain/SRP complexes [24]. These complexes were then preincubated with the original human sera, or the affinity-purified autoantibodies and their corresponding unbound fractions, and the effect of this preincubation on the targeting of the ribosome/nascent chain/SRP complexes to the ER membrane was assessed. Following the incubation of the pretreated ribosome/nascent chain/SRP preparations with rough microsomes, the membrane fraction was recovered by centrifugation. We noted that preincubation of the ribosome/nascent chain/SRP complexes with sera containing SRP autoantibodies significantly reduced the proportion of nascent IMC-CAT_103 _chains recovered in the membrane fraction, consistent with a blockade at some early stage of the membrane insertion process (data not shown).

**Figure 5:**
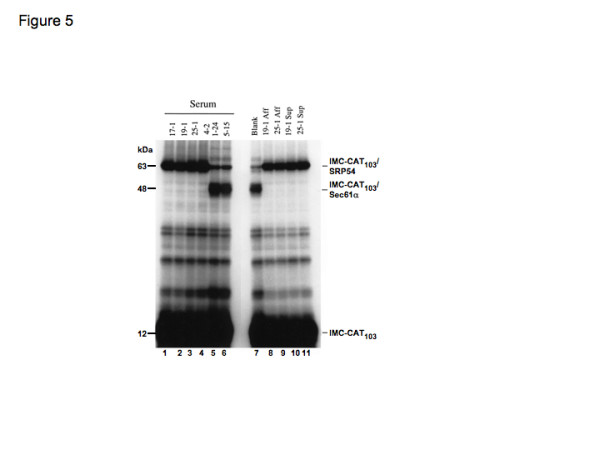
**Autoantibodies against SRP54N and SRP54G domains inhibit release of the signal sequence from SRP54 at the endoplasmic reticulum membrane. **A 103-residue fragment of a model membrane protein derived from regions of the invariant chain of the MHC class II complex, multiple colony-stimulating factor and chloroamphenicol transferase (IMC-CAT_103_) was synthesized *in vitro *as a ^35^S-radiolabelled polypeptide in the presence of SRP and ε-4-(3-trifluoromethyldiazarino) benzoyl-lysine tRNA to yield a stable ribosome/nascent chain/signal recognition particle complex. Cycloheximide was added to prevent further chain elongation and the complex was incubated with sera (lanes 1–6), with no additions (lane 7), with affinity-purified antibodies (Aff, lanes 8 and 9) or with their corresponding unbound fractions (Sup, lanes 10 and 11). Following the addition of endoplasmic reticulum membranes and the activation of the crosslinking reaction by UV irradiation, membrane-targeted nascent chains were isolated by sedimentation through a high-salt/sucrose cushion and the resulting samples were analysed by SDS-PAGE on 10–15% gels and by fluorography. The locations of the IMC-CAT_103 _polypeptide (IMC-CAT_103_), of IMC-CAT_103 _crosslinked to SRP54 (IMC-CAT_103_/SRP54) and of IMC-CAT_103 _crosslinked to Sec61α (IMC-CAT_103_/Sec61α) are indicated.

Following the isolation of the membrane fraction, the association of the IMC-CAT signal sequence with SRP54 was assayed by crosslinking as previously described for PPL (see Figure 4a). In the presence of membranes, the release of the signal sequence from SRP54 is usually very efficient and results in an almost complete loss of the ~65 kDa IMC-CAT_103_/SRP54 product (Figure 5, lane 7). The accompanying appearance of a distinct ~48 kDa product (Figure 5, lane 7, IMC-CAT_103_/Sec61α) reflects the transfer of the nascent chain from SRP54 to the Sec61 translocon [31]. When the two human sera lacking anti-SRP autoantibodies were analysed they gave similar results to the control and efficient release of the nascent chain from SRP54, and transfer to the Sec61 complex was seen (Figure 5, lanes 5 and 6). In contrast, for the samples incubated with sera containing anti-SRP autoantibodies, the ER membrane-associated nascent chains remained bound to SRP54 and no release to the Sec61 complex was seen (Figure 5, lanes 1 to 4). Similar results were seen with the IgG fractions and Fab fragments prepared from these six human sera (data not shown).

When the two affinity-purified antibody preparations were analysed in the same assay, they were also found to completely block the membrane-dependent release of SRP54 from the nascent IMC-CAT chains and only crosslinking to SRP54 was apparent (Figure 5, lanes 8 and 9). Likewise, the two accompanying unbound fractions resulting from the affinity purification of SRP54N domain-specific autoantibodies also completely blocked the release of SRP54 from the nascent IMC-CAT chains (Figure 5, lanes 10 and 11). Affinity-purified autoantibodies specific for the SRP54N region (Figure 5, lanes 8 and 9) and sera containing autoantibodies recognizing the SRP54G domain can therefore inhibit the SRP receptor-mediated release of SRP from an ER signal sequence.

The SRP receptor shows both sequence and structural homology to the N-domain and G-domain of SRP5 [32,33]. It was therefore possible that the autoantibodies bound directly to the SRP receptor and thereby interfered with the release of SRP54 from signal sequences. We found no evidence for any interaction of the anti-SRP autoantibodies with the SRP receptor by immunoprecipitation or immunoblotting, however, and the preincubation of ER membranes with autoantibodies had no effect on protein targeting or translocation, confirming the absence of any function-blocking effect on membrane components (data not shown). We therefore conclude that the inhibitory effect of the anti-SRP autoantibodies on protein targeting is due to their direct interaction with SRP, and primarily with SRP54.

## Discussion

Human autoantibodies that react with SRP have been previously described and shown to immunoprecipitate the 7SL SRP RNA component and to react strongly with SRP54 on immunoblots [12-14]. Anti-SRP autoantibodies are part of a family of myositis-specific antibodies that are directed primarily against conserved conformational epitopes on cytoplasmic components involved in protein synthesis [10]. A unique feature of a number of myositis-specific autoantibodies is that they, in contrast to animal antisera raised to the same proteins, have been found to inhibit the functions of their respective autoantigens [11]. We therefore investigated the effects of anti-SRP autoantibodies on SRP function *in vitro*.

Sera from polymyositis patients containing anti-SRP autoantibodies specifically inhibited the *in vitro *translocation of the secretory protein PPL into the ER. Both IgG fractions and Fab fragments had the same effect, suggesting that a wide-ranging steric inhibition was not occurring. We concluded that one or more anti-SRP autoantibodies present in these sera could block the ER targeting function of SRP. Since SRP54 plays a pivotal role in this process [3,4], and since anti-SRP positive sera were known to contain autoantibodies recognizing SRP54 [15], we focused on the antiSRP54-specific reactivity of these autoantisera.

**Figure 6:**
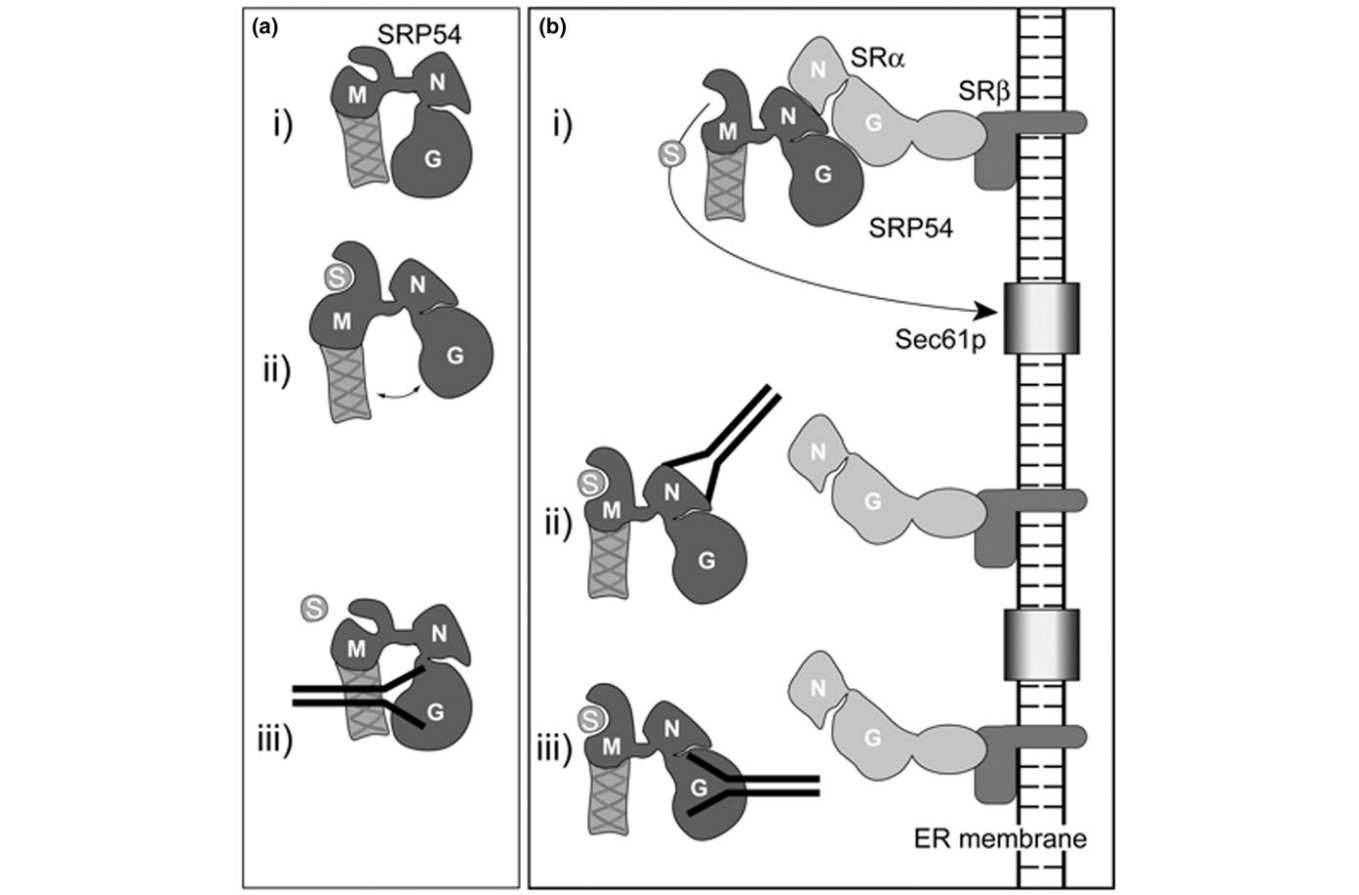
**Schematic showing the effect of subdomain-specific autoantibodies on SRP54 function. (a) **Autoantibodies against the SRP54G domain inhibit signal-sequence binding. Two conformations for SRP54 have been proposed: one where the signal sequence (S) binding groove of the SRP54M domain is closed (i) and the second where it is open (ii). Autoantibodies directed to the G-domain of SRP54 may prevent the conformational change required to open the signal-sequence binding pocket (iii) [44]. **(b) **Autoantibodies against the SRP54N or SRP54G domain prevent signal-sequence release from SRP54 and membrane insertion. When SRP54 interacts with the SRP receptor (SR) at the endoplasmic reticulum (ER) membrane, the signal sequence is released from the M-domain and inserts into the Sec61p translocon in the ER membrane (i). In the presence of autoantibodies directed against the SRP54N or SRP54G domain, the signal sequence remains bound to SRP54 (ii and iii). Ribosomes have been omitted from the nascent chains for the sake of simplicity. The diagrammatic representation of the SRP54 subunit in this model is based on our current understanding of its three-dimensional structure [9,44] rather than on the simple linear organization of the three domains presented in Figure 2a.

We first mapped the region(s) of SRP54 that the anti-SRP positive human sera recognized and found that all four of the sera tested (4-2, 17-1, 19-1 and 25-1) contained autoantibodies that recognized an epitope(s) located within the SRP54G domain. Both sera 19-1 and 25-1 also contained a distinct reactivity towards the SRP54N domain. The SRP54N and SRP54G domains are primarily involved in binding to the SRP receptor (cf. Figure 6b), although these regions may indirectly influence signal-sequence binding [34-36]. None of the sera recognized epitopes in the SRP54M domain, the region of the protein that binds to ER targeting signals [3,4].

Autoantibodies specific for an N-terminal region of SRP54, including the N-domain and part of the G-domain, were affinity-purified from sera 19-1 and 25-1 and were analysed for their effects upon SRP function. We found that both the affinity-purified material, representing autoantibodies specific for the SRP54N domain, and the unbound fraction, which included the activity recognizing the SRP54G domain, blocked PPL translocation in a cell-free system. Autoantibody binding to both of these regions could therefore block SRP-mediated targeting to the ER.

Protein translocation across the ER membrane requires signal-sequence binding to SRP54, followed by targeting of the SRP/nascent secretory protein/ribosome complex to the ER membrane [3]. We found that all of the anti-SRP autoantisera interfered with the binding of SRP54 to the PPL signal sequence when this process was examined directly by photo-crosslinking [25]. While the affinity-purified antibodies specific for the SRP54 N-terminal region had no effect, the unbound antibodies recovered during the purification displayed a strong inhibition of SRP54 binding. We conclude that the binding of SRP54G domain-specific autoantibodies inhibits the ability of SRP to bind to the signal sequence of PPL (Figure 6a, iii). We cannot, however, formally exclude the possibility that this unbound antibody fraction also contains activities that inhibit the function of other SRP subunits, such as the SRP 68 kDa and SRP 72 kDa proteins [37].

Having established that anti-SRP autoantibodies block signal-sequence binding, we investigated their effect upon the SRP-dependent targeting [38]. To study this process we used the model membrane protein IMC-CAT, which contains a signal-anchor sequence that binds to the SRP54 subunit and, upon SRP receptor-mediated delivery to the ER membrane, is subsequently transferred to the Sec61 translocon for membrane integration [24,31] (Figure 6b, i). All four anti-SRP sera completely blocked the transfer of nascent IMC-CAT chains from SRP54 to the Sec61 complex, and the affinity-purified, SRP54N domain-specific antibodies displayed the same level of inhibition as the crude sera. Thus, in contrast to the effects upon signal-sequence binding, autoantibodies specific for both the SRP54N and SRP54G domains can block SRP receptor-mediated targeting to the ER membrane (Figure 6b, ii and 6b, iii). This is entirely consistent with our current understanding of the pivotal role played by the SRP54N and SRP54G domains in the interaction of SRP with the SRP receptor [39,40] (Figure 6b, i). Whether the effect of the anti-SRP autoantibodies is simply to prevent the authentic binding of SRP54 and the SRP receptor, or whether their binding compromises a specific function such as GTP hydrolysis, remains to be established.

## Conclusion

Autoantibodies have frequently been shown to inhibit the function of their target antigens; hence, anti-Sm autoantibodies specifically inhibit mRNA splicing [41] while anti-PCNA autoantibodies inhibit DNA replication [42]. This study provides an example of myositis autoantibodies that, unlike antibodies raised in animals to the same protein, act to inhibit the function of their target autoantigen [11]. A previous study of myositis autoantibody inhibition of autoantigen function found that all six of the anti-aminoacyl-tRNA synthetase antibodies that were tested inhibited enzymatic activity [43].

The reasons for the clear functional differences between spontaneously arising human autoantibodies and animal antibodies raised by immunization with the same protein remain unclear. The findings that myositis autoantibodies arise months prior to clinical disease onset, are antigen-driven and isotype-restricted, have stable spectrotypes, bind to conformational rather than linear epitopes and inhibit the function of their targets, however, all suggest inherent differences in how immune systems respond to autoantigens versus injected antigens [10,11]. One possibility is that human autoantibodies result from interactions of immune-targeted autoantigens with specific environmental agents in the context of disease-associated immunogenetic alleles.

Whatever their origin, myositis autoantibodies continue to provide useful tools both for clinical diagnosis and prognosis, and for dissecting the molecular function of their target autoantigens, including the SRP.

## Abbreviations

ER = endoplasmic reticulum; Fab = antigen binding antibody fragment; IMC-CAT = a chimera of the invariant chain of MHC class II complex, multiple colony-stimulating factor and chloroamphenicol transferase; PBS = phosphate-buffered saline; PCR = polymerase chain reaction; PL = prolactin; PPL = preprolactin; SRP = signal recognition particle; SRP54 = 54 kDa subunit of signal recognition particle.

## Competing interests

The authors declare that they have no competing interests.

## Authors' contributions

FWM identified and supplied the anti-SRP-positive sera and relevant controls, and suggested that human autoantibodies may be useful inhibitors of SRP functions. KR, BD and SH designed the experiments, and KR and SH performed the experiments. All authors contributed to the writing of the manuscript.
